# Cancer Immunotherapy for Immunocompromised Patients: An Often Ignored, yet Vital Puzzle

**DOI:** 10.4103/2666-2345.278414

**Published:** 2020-03-31

**Authors:** Jyoti Bajpai

**Affiliations:** 1 Department of Medical Oncology, Tata Memorial Centre, Homi Bhabha National Institute, Mumbai, Maharashtra, India

Dear Editor,

I have read the recent issue of the *Journal of Immunotherapy and Precision Oncology*, edited by Dr. Joud Hajjar, with immense enthusiasm. It describes “Cancer immunotherapy in immunocompromised patients: An often ignored, yet very vital puzzle!”[1]

These immunocompromised patients have a strikingly high susceptibility to develop a multitude of difficult-to-treat cancers.[2] I wish to expand on the imminent challenges and their pertinency in real-world practice.

In the last decade, immunotherapy, especially immune checkpoint inhibitors (ICIs), has revolutionized the cancer care. Cancer armamentarium is widened with the advent of ICI and the current oncologic indications of ICI encompass melanoma, lung cancer, genitourinary cancer, breast cancer, head and neck cancer, Merkel cell carcinoma, and others.

Notably, immunotherapeutic drugs are different from existing therapies in the mechanism of action, and there is a lack of cross-resistance. The recent evidence further emphasizes that immune evasion is important for carcinogenesis and certain arduous cancers preferentially develop in immunocompromised hosts.[2,3] The potential factors affecting this are the specific aspects of immune deficiency, the duration of immunosuppression, the immunomodulatory agents used, and the differences in patient populations. However, a more appealing and unique facet is the labyrinthine relationship between immunosuppression and cancer and their co-action with ICI agents driving the antitumor immune response.[2–4] Despite increasing indications and usage, we still have limited knowledge regarding the efficacy and safety in this exclusive immunosuppressed population as these patients are excluded from the clinical trials. Nonetheless, retrospective studies reported safe use of ICI in immunocompromised situations such as HIV infection or autoimmune disorders.[2,4,5] Some of the recent studies showed that ICIs are both safe and efficacious in HIV-positive patients with various malignancies, including Kaposi sarcoma, nonsmall cell lung cancer, melanoma, Merkel cell carcinoma, and Hodgkin's lymphoma.[2,4]

Clinical trials are underway to provide robust answers for the safety and efficacy of ICI in this population. Per contra, literature on ICI use in transplant recipients is discordant, where there is a real threat of possibly lethal-transplant rejections as high as 40%, physicians should be wary using ICI in this unique, data-free zone.[6]

Another similar population is patients on low-dose steroids for a variety of clinical conditions such as respiratory illnesses, fatigue, or brain metastasis. There is an argument against early or baseline use of steroids with ICI, which emerged from retrospective lung cancer cohorts.[7,8] In those cases, it is reasonable switching to alternative medications or decreasing corticosteroid dose (<10 mg) if feasible, recognizing that patients who are receiving steroids typically have more aggressive and extensive disease, which represents a possible confounding effect when interpreting these data. In contrast, late use of steroids in the treatment course may confer improved outcomes, perhaps reflecting the evidence linking immune-related adverse events and efficacy of ICI.[9,10]

I highly commend Dr. Hajjar for lighting up this crucial and often ignored controversy![1]
